# Meetings of Societies

**Published:** 1935

**Authors:** 


### Meetings of Societies

### Bristol Medico-Chirurgical Society

SESSION 1934–35.

THE fourth meeting of the Session was held in the Physiological Lecture Theatre of the University on Wednesday, 9th January, 1935. The President, Mr. A. Rendle Short, was in the chair, and fifty members were present.

Dr. A. L. Taylor and Dr. T. B. Wansbrough showed pathological specimens and skiagrams respectively.

The minutes of the previous meeting were read and confirmed, and Dr. A. L. Taylor, at the request of the President, described the specimens which he had shown.

Dr. H. H. Carleton then read a paper entitled "**Habit and Illness,**" which is published on p. 41 of this issue, and he was congratulated by the President. In the discussion which followed the following spoke: The President, Dr. Frank Bodman, Mr. Adams, Dr. Glenny, Dr. Symes and Dr. Alexander and Dr. Carleton replied.

The fifth meeting of the Session was held in the Physiological Lecture Theatre of the University at 8.30 p.m. on Wednesday, 13th February, 1935. The President, Mr. A. Rendle Short, was in the chair, and thirty-two members were present.

Dr. A. D. Fraser showed pathological specimens.

The minutes of the previous meeting were read and confirmed.

The Secretary then read a communication from Dr. Hastings Moore, announcing that there would be a meeting of the Medical Dramatic Club on 21st February in the Physiological Lecture Theatre.

Mr. A. E. Iles and Mr. G. R. Scarff then read a paper on the "**Diseases of the Lachrymal Sac,**" and the following took part in the discussion which followed: The President, Mr. E. Watson-Williams, Mr. Garden, Mr. Wright, Mr. Walbank, Mr. James, Dr. Gee and Mr. Shepherd, and Mr. Iles and Mr. Scarff replied.

---

The sixth meeting of the Session was held in the Physiological Lecture Theatre of the University on Wednesday, 13th March, at 8.30 p.m. The President, Mr. A. Rendle Short, was in the Chair, and there were ninety-four members present.

Dr. A. L. Taylor and Dr. G. B. Bush showed Pathological Specimens and Skiagrams respectively.

The minutes of the previous meeting were read and confirmed, and the President then introduced Professor Miles H. Phillips, who gave an address on "**Men-Midwives—Past and Future.**"

The President, congratulated Professor Miles Phillips and thanked him for his address, and in the short discussion which followed the President, Professor Drew Smythe, Mr. E. Watson-Williams, Dr. Maxwell and Dr. Alexander spoke, and Professor Miles Phillips replied.

A vote of thanks to Professor Miles Phillips was proposed by Dr. Nixon and seconded by Professor Drew Smythe, and this was carried with acclamation.

